# *Plasmodium yoelii* as a model for malaria: insights into pathogenesis, drug resistance, and vaccine development

**DOI:** 10.1007/s11033-025-10318-4

**Published:** 2025-02-05

**Authors:** Oluwatobi Otun, Ikechukwu Achilonu

**Affiliations:** Protein Structure-Function Unit, Department of Molecular and Cell Biology, University of Witwatersrand, Johannesburg, South Africa

**Keywords:** Drug resistance, Host-parasite interactions, Malaria pathogenesis, *Plasmodium yoelii*, Vaccine development

## Abstract

Malaria continues to pose a serious global health threat, with rising drug resistance making treatment more difficult. Consequently, *Plasmodium yoelii*, a rodent malaria parasite, has become an invaluable model for studying the malaria parasite’s complex biology, particularly host-parasite interactions and drug resistance mechanisms. Originating from African rodents, *P. yoelii* presents unique genetic diversity, mirroring critical aspects of human malaria and allowing for detailed research on the parasite’s pre-erythrocytic and erythrocytic stages. This review introduces a novel perspective by comprehensively examining *P. yoelii* from multiple angles, including its origin, genetic composition, and contributions to vaccine development and drug efficacy studies. It highlights the significance of *P. yoelii,* revealing new insights into immune evasion, pathogenesis, and disease severity, which are critical for developing future malaria interventions. Furthermore, this review addresses the challenges associated with *P. yoelii* as a model, such as differences in host immune responses between rodents and humans. Despite these hurdles, the research using *P. yoelii* offers promising directions for combating malaria. This novel synthesis of *P. yoelii* studies underscores its essential role in advancing malaria research, particularly in understanding drug resistance and uncovering potential therapeutic and vaccine targets.

**Graphical Abstract:**

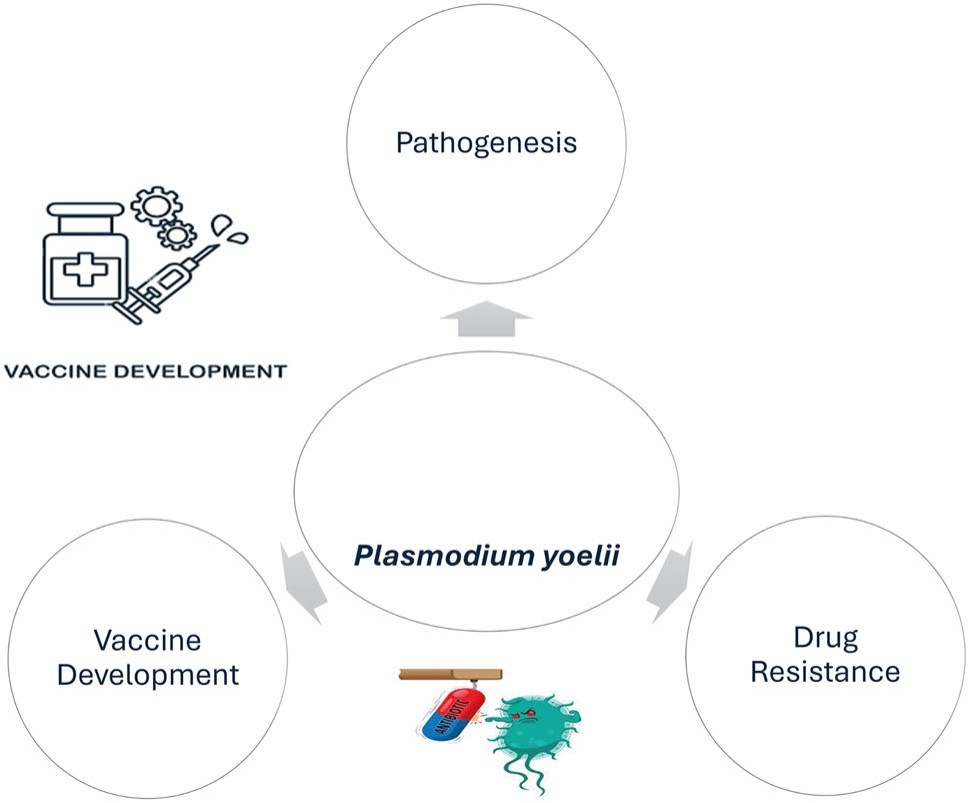

## Introduction

Malaria is a global health problem that causes a high rate of morbidity and death, especially in pregnant women and young children [1]. It is known that Plasmodium genus protozoan parasites cause this disease, and the female mosquitoes (*Plasmodium falciparum)* are the primary transmission vector [2], especially in sub-Saharan Africa, where it is responsible for most severe cases and deaths [3]. Unfortunately, drug-resistant Plasmodium strains and shifting ecological factors continue to make control efforts more difficult despite continuous international efforts to combat malaria, which include the use of antimalarial medications and vector control techniques [4]. This underscores the need for novel approaches to malaria research.

Consequently, *P. yoelii* is a parasite that causes malaria in rodents, and it has become crucial to this knowledge because it has become a model organism for researching the intricate life cycle, immunological reactions, and pathophysiology of malaria [5]. *P. yoelii* was first isolated from wild rodents in Africa and is now a valuable model for investigating malaria parasite growth in liver cells and vectors [6]. This is mainly because of its unique features, including its shorter life cycle, well-characterised genetic background, and the capacity to conduct studies impractical in human-infecting species[5, 6]. Thus, understanding malaria biology, host-parasite interactions, and the processes causing medication resistance has been improved due to research employing *P. yoelii* as a research model.

This review explores the multifaceted role of *P. yoelii* in malaria research, covering its origins, genetic composition, host-parasite interactions, and contributions to vaccine and drug development. It also emphasises *P. yoelii’s* vital function as a model for furthering malaria research and creating cutting-edge preventative and treatment methods by combining these elements. By examining the challenges and advancements in understanding this model organism, we aim to effectively enhance strategies for combating malaria.

## The origins of *Plasmodium yoelii* reveals its foundational role in malaria research

*Plasmodium yoelii’s* history dates to 1948 when Dr David Walliker and Dr Richard Carter of the University of Edinburgh identified *P. yoelii*, the first rodent malaria parasite, from an African thicket rat *(Grammomys surdaster*) in the Democratic Republic of the Congo’s Kisanga province. This research established the rodent-to-mosquito lifecycle of *P. yoelii* when its sporozoites were effectively transferred to white mice by Anopheles mosquitoes [7]. However, over the next two decades, additional rodent malaria parasites were discovered across Africa. Plasmodium species and subspecies were found in various nations, according to [7], which depicts the geographic distribution and variety of rodent malaria parasites across Africa. *P. yoelii nigeriensis* and *P. vinckei brucechwatti* have been found in Nigeria, whilst *P. yoelii cameronensis*, *P. vinckei baforti*, and *P. chabaudi esakensis* are found in Cameroon. The species *P. chabaudi adami*, *P. vinckei lentum*, and P*. yoelii killicki* are found in Brazzaville, Republic of Congo. Similarly, *P. chabaudi chabaudi, P. vinckei petteri*, and *P. yoelii yoelii* were found in the Central African Republic. Finally, *P. vinckei vinckei* and *P. berghei spp* were found in the Democratic Republic of Congo.

Thus, the identification of *P. vinckei* in 1952, and 1966, *P. yoelii yoelii* was isolated from *Thamnomys rutilans* in West and Central Africa and was later renamed *P. yoelii* in honour of Professor Meir Yoeli, who developed the *P. yoelii’s* cyclical transmission techniques [8]. This species was further classified into three subspecies: *P. yoelii yoelii*, *P. yoelii killicki*, and *P. yoelii nigeriensis,* based on distinct geographical distributions and enzyme profiles [9]. However, the known vector for *P. vivax*, the female Anopheles mosquito, is thought to be a possible carrier for *P. yoelii* transmission, even though the natural vectors of the parasite are yet unknown [5].

Interestingly, the 1970s saw a turning point in categorising *P. yoelii* based on the research on isoenzyme variation, revealing the genetic differences among rodent malaria species [7]. Furthermore, the pathophysiology of malaria has been greatly aided by rodent malaria parasites, particularly in the study of sporozoite movement and the formation of the pre-erythrocytic stage in hepatocytes. Effective separation of liver stage-infected hepatocytes from the mouse host was made possible by the availability of *P. yoelii* expressing GFP, which offers information on the transcriptome and proteomic profiles of the liver stage development [10].

Lastly, using electrophoresis, researchers evaluated the genetic diversity of *P. yoelii* and its subspecies. Changes in enzyme activity established the taxonomic framework that allowed for the efficient study of *P. yoelii* and revealed information about the evolutionary connections between the isolates [2]. This basis allowed scientists to use *P. yoelii* as a model organism to study drug resistance mechanisms and host-parasite interactions, among other aspects of malaria biology. *P. yoelii* was a vital tool for investigating the intricacies of malaria pathophysiology and its resilience to laboratory settings, making it a reliable research model.

## Exploring the diversity: classification and subspecies of *Plasmodium yoelii*

Precise classification is essential in malaria research to identify different species and their subspecies, which might differ significantly in pathogenicity, host interactions, and ecological adaptations. Six known subspecies of *P. yoelii*, each with distinct traits and contributions to malaria research, make up the taxonomy of this very noteworthy parasite. Table 1 gives an outline of *P. yoelii’s* taxonomy.

**Table 1:** Taxonomy of *Plasmodium yoelii*. Source: [11]

Classification Level	Taxonomic Rank
Domain	Eukarya
Kingdom	Plantae
Phylum	Apicomplexa
Class	Aconoidasida
Order	Haemosporida
Family	Plasmodiidae
Genus	Plasmodium
Subgenus	Vinckeia
Species	*Plasmodium yoelii*
Subspecies	*Plasmodium yoelii 17* *Plasmodium yoelii 17X* *Plasmodium yoelii killicki* *Plasmodium yoelii nigeriensis* *Plasmodium yoelii YM* *Plasmodium yoelii yoelii*

There are six known subspecies of *P. yoelii* in the NCBI database that exist [11]. Moreover, each has distinctive characteristics that add variety and importance to malaria research, as shown in Table 2

**Table 2:** Distinctive characteristics and research importance of *Plasmodium yoelii* subspecies

Subspecies	Distinctive Characteristics	Importance in Malaria Research	References
*Plasmodium yoelii 17*	The original research strain has mild virulence	It is a reference strain in many experimental studies and is often used for baseline comparisons	[12]
*Plasmodium yoelii 17X*	Clonal line derived from *P. yoelii17*; non-lethal strain	Utilised to study immune responses and host–pathogen interactions, especially in cases of non-lethal infections. Provides insight into the dynamics of chronic and mild malaria infections	[13]
*Plasmodium yoelii killicki*	Found in rodent hosts in Central Africa, it exhibits moderate genetic variation compared to other strains	It is essential to study genetic diversity within P. yoelii subspecies and their adaptation to different rodent hosts	[14]
*Plasmodium yoelii nigeriensis*	Displays significant genetic variation from other *P. yoelii* strains, highly virulent	It is crucial for understanding genetic factors that drive virulence and the development of drug resistance. Its genomic diversity makes it a key target for comparative genomic studies	[15]
*Plasmodium yoelii YM*	A lethal strain, fast-replicating with high virulence, is often used in experimental studies involving severe malaria	Key for studying mechanisms of severe malaria, including immune evasion, parasite growth, and host mortality. Frequently used in vaccine development and drug efficacy studies due to its lethal nature	[16]
*Plasmodium yoelii yoelii*	Includes both lethal and non-lethal strains; known for genetic stability and reproducibility in research	It provides a model for genetic studies and gene editing, allowing researchers to study both mild and severe malaria phenotypes	[17]

This table highlights the diversity within *Plasmodium yoelii* subspecies, each playing a crucial role in malaria research, particularly in understanding parasite genetics, virulence, and treatment responses

## Genetic composition of *Plasmodium yoelii* unravels its infection dynamics

*Plasmodium yoelii*, like its closely related species *P. berghei* and *P. chabaudi*, displays many behaviours and genotypes that make it a model organism in malaria research [18]. Distinct physical and developmental characteristics enable the *P. yoelii* group of parasites to be easily distinguished from the *P. berghei* group. For instance, the 14 chromosomes around the 21.9 Mb haploid genome of *P. yoelii* are similar to other malaria parasites infecting humans and rodents [48]. In comparison, the genome of P*. yoelii nigeriensis* N67 is somewhat smaller at about 21.3 Mb, with 5383 predicted genes and 121 contigs with around one SNP every 50 bp sequence, strains like *P. yoelii* YM/17X and *P. yoelii. nigeriensis* N67 vary significantly in their genomes. Only eight genes separate the genomes of the P. *yoelii* —yoelii YM and 17X strains, which are otherwise almost similar [19]. Variable gene families, such as the yir and fam gene families, are responsible for this genome variety and help explain the parasite’s virulence and growth flexibility [48]. Thus, genome sequencing and annotation of *P. yoelii* strains have made it possible to understand genetic variation and enhance functional studies, particularly those examining malaria pathogenesis and host-parasite interactions.

Moreover, *P. yoelii* selectively invades both mature and immature erythrocytes and is known to cause synchronous blood-stage infections. It usually generates an average of 6–8 merozoites per schizont. *P. yoelii* expressing GFP made it possible to isolate liver stage-infected hepatocytes from the rodent host with efficiency, which offers information on the transcriptome and proteomic profiles of the liver stage development [10].

Although several *P. yoeli*i subspecies have similar physical traits, and isolates might differ significantly in terms of virulence and infection history [20]. Variations in parasitemia levels and host responses reported in laboratory investigations demonstrate the genetic heterogeneity within the *P. yoelii* population; this could be due to variations depending on the environment, especially temperature, which influences the mosquito vector’s pace of growth [21].

Furthermore, significant isoenzyme differences across isolates were found in early investigations of the *P. yoelii genetic* variety, suggesting a degree of genetic variability that molecular methods have now validated [22]. The genetic diversity of the *P. yoelii* genome has been highlighted by the discovery of thousands of single nucleotide polymorphisms (SNPs) in recent genomic investigations [23]. With distinct genetic markers that might affect host interactions and treatment responses, *P. yoelii exhibits* significant divergence from its closest relatives. The evolutionary dynamics of *P. yoelii* are influenced by structural differences and synteny modifications found in its genome [24]. *P. yoelii* is now a crucial model for researching malaria biology since its whole genome sequencing studies have identified specific gene families linked to virulence and immune evasion tactics [19]. These discoveries open up new study possibilities for comprehending the intricacies of malaria pathophysiology and resistance mechanisms and clarifying the genetic landscape of *P. yoelii*.

## Life stages of *Plasmodium yoelii*: pre-erythrocytic and erythrocytic research

**Fig. 1:**
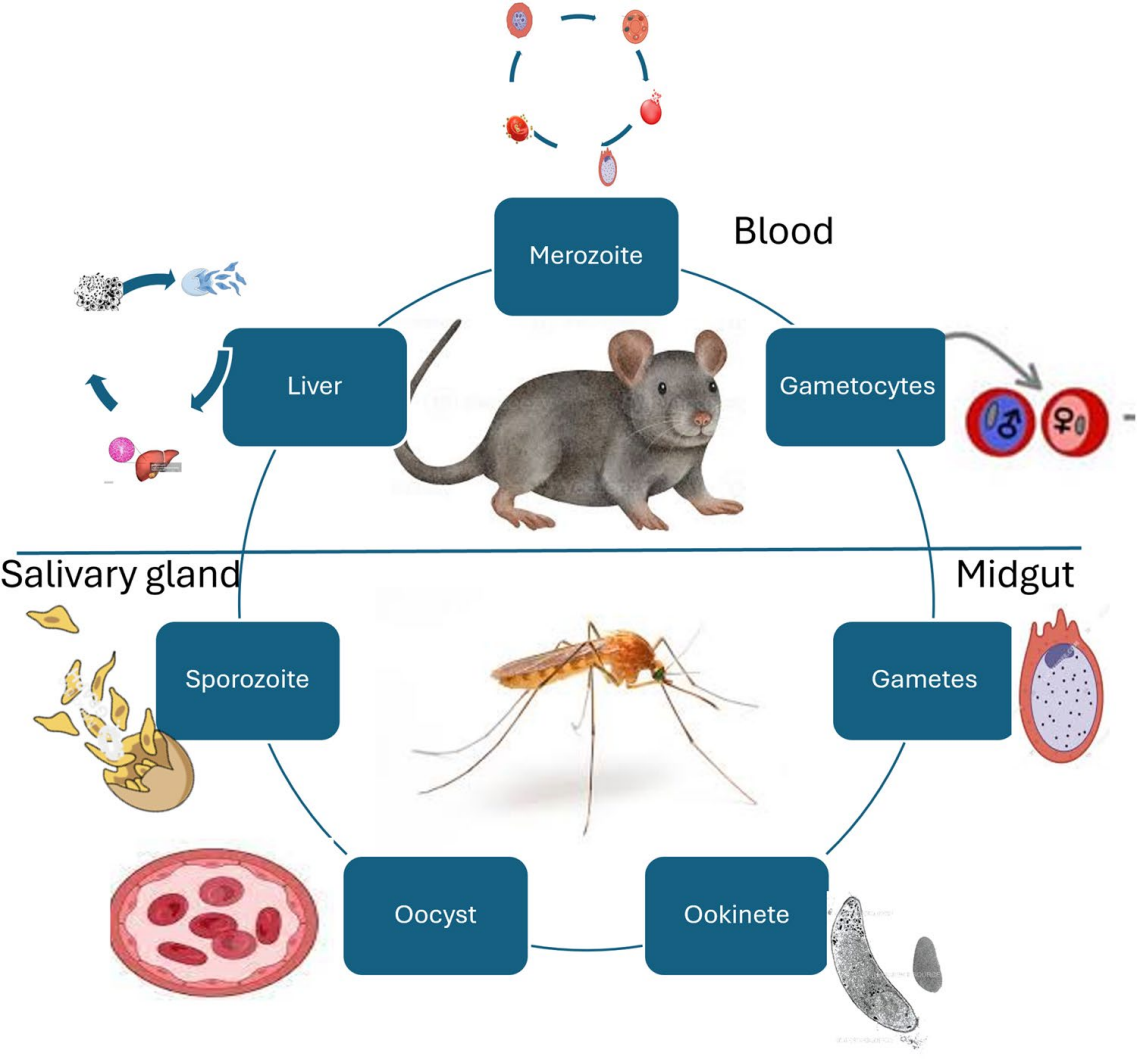
The life cycle of *P. yoelii*

*Plasmodium yoelii* serves as an essential model for studying both the pre-erythrocytic and erythrocytic stages of malaria infection, providing valuable insights into the parasite’s complex life cycle (Fig. 1) [25, 26]. Understanding these stages is crucial for developing targeted interventions to interrupt the parasite’s development and improve treatment strategies [27].

Figure 1 shows the *Plasmodium yoelii* life cycle with important stages in the rodent and mosquito host. It shows the liver stage (infection of the hepatocyte), blood stage (replication of the merozoite), and mosquito stage (sporogony), marking parasite development and transmission between the hosts. The life cycle of *P. yoelii* stages is quite different from other Plasmodium parasites, such as *P. berghei*, *P. falciparum*, and *P. vivax* and will affect their applications in model studies. The mosquito stage of *P. yoelii* requires about 10–14 days to mature into sporozoites, as it also does in *P. berghei*, but less than that of *P. falciparum* (14–16 days). The *P. yoelii* liver cycle takes 40–48 h to complete, as does *P. berghei*, but significantly more quickly than *P. vivax* (6–8 days). The *P. yoelii* erythrocyte cycle is recycled every 24 h, compared to *P. falciparum* (48 h) and *P. malariae* (72 h). Each of these variations affects disease severity, host immune response, and drug sensitivity among species.

In the pre-erythrocytic stage, *P. yoelii* sporozoites are injected into the host during a mosquito’s blood meal. Once in the liver, these sporozoites invade hepatocytes and undergo asexual replication, eventually forming schizonts that release merozoites into the bloodstream [28]. Research has demonstrated that studying this phase using *P. yoelii can* reveal mechanisms of liver-stage infection and host immune responses. For instance, it has been shown that vaccination with irradiated sporozoites can confer protection by prompting robust immune responses that target liver-stage antigens, which could lead to new strategies for preventing malaria before it spreads to the bloodstream [29]. Additionally, according to a study on the comparative transcriptional analysis of pre-erythrocytic stages, including radiation-attenuated sporozoites (RAS), wild-type sporozoites (wtSPZ), and liver-stage parasites (24-h and 48-h stages post-infection). The study revealed over 1100 *Plasmodium* genes with differential expression compared to the mixed blood stages. As the parasite progressed from the sporozoite to the liver stage, its transcriptional profile became increasingly similar to the blood-stage parasites. Notably, the gene expression patterns between RAS and wtSPZ were nearly identical, with the most significant differences occurring between sporozoites and liver-stage parasites. These findings may lead to the discovery of novel drug and vaccine targets by further characterising the biological mechanisms of the pre-erythrocytic stages [25].

Whereas, in the erythrocytic stage, the released merozoites invade red blood cells, leading to the clinical manifestations of malaria. This stage is critical for understanding disease pathology and the immune response to malaria [30]. Using *P. yoelii*, researchers have investigated how the immune system responds to blood-stage infections, identifying key antigens and understanding parasitemia dynamics. Howells et al. [31], compared the sensitivities of pre-erythrocytic and erythrocytic stages of drug-sensitive and drug-resistant strains of *P yoelii* to cycloguanil and pyrimethamine. In both strains, the pre-erythrocytic stages showed higher sensitivity to these drugs than the erythrocytic stages. In the drug-resistant strain, resistance in erythrocytic stages was mirrored by a decreased sensitivity in the pre-erythrocytic stages. These studies have shown that targeting the erythrocytic stage can lead to effective treatments that disrupt parasite growth and reduce the severity of disease symptoms.

Overall, the ability to study both the pre-erythrocytic and erythrocytic stages of malaria infection in *P. yoelii* enhances our understanding of the parasite’s life cycle and informs the development of interventions that can effectively target different stages. This research is vital for devising comprehensive strategies for optimal drug timing for malaria treatment. This understanding will help customise treatment plans, improving therapeutic outcomes and reducing resistance risks by targeting parasites during their most susceptible stages.

## Understanding host-parasite interaction studies with *Plasmodium yoelii*

*Plasmodium yoelii* has become a cornerstone for studying host-parasite interactions in malaria research, shedding light on the complex dynamics between the malaria parasite and its rodent hosts [17, 19]. This model is beneficial for understanding how the parasite invades red blood cells and how it evades the host’s immune system during various stages of infection [12, 14].

One of the critical research areas involves the mechanisms by which *P. yoelii* merozoites invade erythrocytes [12]. Studies have shown that the interaction between the parasite’s surface proteins and receptors on red blood cells is crucial for successful invasion [32]. For instance, a study by Grüber et al. [33] highlighted that specific adhesins, like the reticulocyte-binding protein homolog, play a significant role in this process. They confirmed that the invasion of host red blood cells by *P yoelii* is a critical step for the parasite’s survival, and it presents a prime target for antimalarial interventions. Their study identified the erythrocyte binding domain (EBD) of a reticulocyte binding protein, Py235, which directly binds to red blood cells. Using small-angle X-ray scattering, they were able to map the low-resolution structure of Py235’s EBD. Structural conservation was noted compared to other *Plasmodium* receptor binding domains, offering valuable insights for developing strategies to block these essential receptor-ligand interactions. This research demonstrated that inhibiting these proteins could reduce the parasite’s ability to attach to and invade erythrocytes, offering potential avenues for therapeutic interventions [33].

Additionally, *P. yoelii* provides insights into how the parasite interacts with the host’s immune system throughout the infection [34]. According to Peng et al., [26], a novel role of *P. yoelii* erythrocyte-binding-like (EBL) proteins in modulating host immune responses and influencing disease severity was discovered. A specific amino acid substitution (C741Y) in the PyEBL protein impacted parasite growth, host survival, and the surface structure of infected red blood cells (iRBCs). This alteration increased phosphatidylserine exposure, enhancing the phagocytosis of iRBCs via the PS-CD36 pathway and triggering type I interferon signalling and T-cell differentiation in the host. While the mutation did not affect red blood cell invasion, it provided valuable insights into host-parasite interactions and potential disease control strategies.

Research has also shown that the immune response can be protective and pathogenic, as influenced by the parasite’s ability to modulate immune pathways [35]. For instance, Azcárate et al. [21] studied a *P yoelii* mouse model that mimics the heterogeneous nature of malaria infection, offering insights into different immunological outcomes. Three distinct disease stages were observed in a non-congenic ICR strain: early fatal, late fatal, and self-resolving infections. Mice with high parasitemia and early death exhibited increased monocytes, dendritic cells, immature B cells, and an early expansion of CD4 + CD25 high T cells expressing Foxp3. In contrast, survivors demonstrated limited cytokine release and a stable innate immune response. Long-term immunity in survivors was marked by an expansion of activated T cells and class-switched B cells, highlighting potential immune markers for prognosis. Also, Orengo et al. [36] found that *P. yoelii* can induce a robust inflammatory response by activating dendritic cells and macrophages, which can contribute to disease pathology. Understanding these immune responses is crucial for developing effective vaccines and therapies.

Lastly, the research involving *P. yoelii* helps clarify how genetic variations in parasites and hosts affect susceptibility to malaria [37]. Research on *P. yoelii* infections in inbred and H-2 congenic mouse strains reveals that resistance and susceptibility to malaria vary depending on the host’s genetic background and the parasite strain. DBA/2 and B10.D2 mice, resistant to non-lethal isolates, were highly susceptible to lethal strains, while B6 and B10 mice showed the opposite pattern. H-2 genes partly influence this resistance reversal, but genes outside this complex also play a role. Interestingly, a robust immune response in B6 mice was linked to increased susceptibility, suggesting that immune responses can drive vulnerability during early infection stages rather than protection [37]. Thus, by studying different strains of *P. yoelii* in various mouse models, scientists can investigate the genetic factors that influence the outcome of infection and host resistance mechanisms. Based on this hypothesis, a comparative study revealed that specific genetic backgrounds in mice could lead to different immune responses to *P. yoelii* infection, influencing the severity of the disease [38]. Therefore, *P. yoelii* is invaluable for investigating host-parasite interactions, enhancing our understanding of the invasion process and the host immune response. This knowledge is crucial for developing new therapeutic and preventive measures against malaria, ultimately contributing to the global fight against this devastating disease.

## Studying pathogenesis and disease severity using *Plasmodium yoelii*

This variability of virulence of the different strains of *P. yoelii* allows researchers to explore the intricate mechanisms leading to severe malaria and understand the factors contributing to disease severity [39]. This is because they exhibit distinct pathogenic profiles, which mimic the variability seen in human malaria infections [40]. For instance, the *P. yoelii yoelii* strain is known to cause more severe disease than the less virulent *P. yoelii nigeriensis* [26]. This differential virulence provides an excellent model for investigating how specific genetic and biological factors influence disease outcomes.

Research by Omer et al. [41] found that the impact of malaria infections depends significantly on where TGF-β is produced and when. Mice infected with the nonlethal *P. yoelii* strain (Py17X) start producing TGF-β five days after infection, which aligns with a drop in parasitemia and a decrease in TNF-α levels, ultimately leading to recovery. In contrast, the lethal strain (Py17XL) causes a spike in TGF-β within 24 h, impairing immune responses and resulting in 100% mortality. Blocking early TGF-β production during Py17XL infection triggers a rise in IL-10 while simultaneously blocking both TGF-β and IL-10R signalling increases TNF-α and IFN-γ levels, allowing prolonged survival and infection resolution in 40% of these mice. Notably, TGF-β can be triggered from the splenocytes of infected mice in an antigen-specific way. After stimulation with Py17X, TGF-β mainly comes from CD25+ and CD8+ cells, whereas Py17XL significantly prompts TGF-β production from adherent cells. Mice vaccinated against Py17XL show reduced early TGF-β responses and higher IFN-γ and TNF-α levels, leading to quick resolution of subsequent infections. This showed that the more virulent strains induce higher levels of pro-inflammatory cytokines, leading to severe immunopathology in infected hosts.

Furthermore, using *P. yoelii* in experimental models allows scientists to dissect the molecular and cellular mechanisms driving the disease [42]. Researchers can identify critical factors exacerbating malaria pathology by examining how different strains interact with host immune responses. For example, a study by Couper et al. [43] proposed that severe infections from the lethal strain of *P. yoelii* (17XL) might result from an insufficient inflammatory response. Their study compared the adaptive CD4+ T-cell and innate immune responses between the lethal strain (17XL) and the nonlethal strain (17X(NL)). They found that during the initial 7 to 9 days, CD4+ T-cell responses were similar for both strains. Interestingly, RAG−/− mice controlled nonlethal infections as effectively as wild-type mice, suggesting minimal involvement of T and B cells. Depleting monocytes/macrophages also exacerbated parasite growth, revealing an alternative pathway for effector macrophage activation independent of IFN-γ and NK cells. This demonstrated that certain *P. yoelii* strains trigger a more robust inflammatory response, contributing to increased parasite burden and higher mortality rates [43]. These findings underscore the importance of understanding host-parasite interactions in the context of virulence.

Moreover, *P. yoelii* strains can be used to evaluate the effects of genetic factors on disease severity. By performing genetic crosses and creating hybrid strains, researchers can pinpoint specific genes responsible for virulence [26]. This approach was highlighted in a study by Su et al. [44]; the authors identified several candidate genes linked to severe disease outcomes in mice infected with virulent *P. yoelii* strains. Therefore, studying *P yoelii* provides crucial insights into malaria’s pathogenesis and disease severity. By utilising strains with varying virulence, researchers can unravel the complexities of malaria pathology, paving the way for targeted interventions that may improve treatment and prevention strategies for severe malaria in humans.

## Testing drug efficacy and drug resistance in *Plasmodium yoelii*

Due to the ability of *P. yoelii* to replicate the critical aspects of malaria infections in humans, researchers have found it invaluable in preclinical testing, i.e. evaluating the efficacy of antimalarial drugs and screening new drug candidates before advancing to human clinical trials. testing [19, 28]. One key advantage of using P. yoelii is its rapid assessment of drug efficacy; studies often employ this model to screen new compounds against the parasite swiftly. This efficient process is a testament to the model’s effectiveness in drug research. [45]. Several studies often employ this model to rapidly screen new compounds against the parasite. Research led by Srivastava et al. [46] tested many compounds against *P. yoelii* and showed promising results in inhibiting parasite growth, potentially translating to effectiveness against human malaria. This research demonstrated a link between in vitro and in vivo antimalarial activity using MIC, IC50, and IC90 values against both chloroquine-sensitive (3D7) and chloroquine-resistant (K1) strains of *P. falciparum*, as well as *P. yoelii*in vivo activity. Discriminant function analysis revealed a strong correlation between in vitro IC90 values and in vivo curative efficacy (p < 0.001). Compounds that cured mice had IC50 and IC90 values ranging from 3–14 nM and 14–186 nM for the 3D7 strain, respectively. The findings suggest that IC90 values are crucial for predicting the curative activity of new antimalarial molecules [46].

In addition, *P. yoelii* has been used to investigate the mechanisms of action of existing antimalarial drugs. For example, Witkowski et al. [47] explored the effects of artemisinin-based combination therapies (ACTs) on *P. yoelii*. Their study revealed that ACTs effectively reduced parasite burden in infected mice and identified specific stages of the parasite’s lifecycle most susceptible to treatment. Similarly, Ginkgo biloba extract (GBE) was combined with artemisinin to create artemisinin-GBE combination therapy (AGCT) for treating *P. yoelii* infections. AGCT exhibited significant antimalarial effects by reducing infection rates, enhancing blood microcirculation, and modulating the immune response. AGCT suppressed the expression of invasion-related genes, including AMA1, MSP1, and Py01365, thereby hindering merozoite invasion [15]. Thus, combining antimalarial drugs with agents that improve blood circulation could enhance overall treatment efficacy and facilitate recovery [49].

Unfortunately, drug resistance is a significant challenge in malaria treatment. Using *P. yoelii,* researchers can develop and study resistant parasite lines to assess the impact of drug resistance on treatment outcomes. A notable study by Ferrer-Rodríguez et al., [50], demonstrated that resistant strains of *P. yoelii c*ould be used to evaluate the effectiveness of new compounds in overcoming resistance mechanisms. They examined drug resistance in *P. yoelii* using lines with varying resistance profiles and identified the mdr1 gene (pymdr1). Notably, the artemisinin-resistant *P. yoelii* ART line exhibited a two- to three-fold increase in pymdr1 copy number compared to the non-resistant parental line, with gene expression confirmed at both RNA and protein levels through reverse transcriptase–polymerase chain reaction and Western blot analyses [50].

Investigating drug resistance in malaria is imperative to develop efficient treatment techniques and comprehend the underlying genetic pathways. Due to its relative ease of manipulation in laboratory conditions, *P. yoelii* has been a beneficial model organism for drug resistance research. Researchers can create resistant cells in much less time with *P. yoelii*, usually requiring fewer passes and more straightforward procedures, than with *P. falciparum*, where producing stable drug-resistant lines can take months or even years. Although some studies have shown that different *P. yoelii* strains can become permanently resistant to antimalarial medications, such as artemisinin and chloroquine [1, 49, 50]. For example, many drug-resistant strains of *P. yoelii* have been isolated due to the straightforward process of selecting rodent malaria parasites resistant to antimalarial medications in mice [51]. It should be noted that while some *P. yoelii* parasites are inherently resistant to low-level chloroquine (CQ) [52], other *Plasmodium* Species are susceptible to most antimalarial medications, including pyrimethamine and artemisinin (Table 3) [53]. Table 3 highlights the drug resistance mechanisms across different *Plasmodium* species, comparing their identified resistance genes and mechanisms.

**Table 3:** Comparative analysis of drug resistance mechanisms in *P. yoelii* and other *Plasmodium* species

*Plasmodium* species	Identified resistance genes	Mechanisms of resistance	References
*Plasmodium falciparum*	pfcrt, pfmdr1, pfdhfr	Altered drug targets, reduced drug accumulation	[54–56]
*Plasmodium vivax*	pvmdr1, pvdhfr	Transporter mutations, altered metabolism	[57]
*Plasmodium knowlesi*	pkmdr1	Efflux pumps, reduced susceptibility	[57]
*Plasmodium yoelii*	RhopH1, RhopH3, ApiAP2,	Transport mutations, immune evasion strategies	[58, 59]

Many cloned lines of the rat malaria parasites that show diversity in virulence have been developed thanks to advancements in cloning techniques and the discovery of numerous genetic and biochemical markers. These lines are ideal models for researching disease pathophysiology [20].

Additionally, *P. yoelii* model has been helpful in the investigation of recrudescence occurrences after antimalarial therapy [60, 61]. Research reveals that a developing primary immune response involving B and CD4+T cells is crucial for eliminating *P yoelii* YM parasites in ART-treated mice. However, it is not necessary for initial clearance. The study by Claser et al. [60] highlights the complex interaction between ART and hosts adaptive immunity in eliminating malaria parasites, emphasising the immune system’s role in treatment efficacy [60]. A return of parasitemia may result from the persistence of latent stages of *P. yoelii* following medication delivery, according to research by [62].

This behaviour emphasises how crucial it is to comprehend *P. yoelii*’s life cycle and how it affects methods for treating and controlling malaria. Using *P. yoelii* in drug resistance research helps identify targets for novel therapeutic approaches and advances our knowledge of the genetic basis of resistance. It allows researchers to screen new drug candidates and evaluate the effectiveness of existing treatments. Through these studies, scientists can maximise the experimental and therapeutic advantages of studying *P.yoelii* (Table 4) into drug action and resistance mechanisms, ultimately aiding the fight against malaria.

**Table 4:** Experimental advantages and therapeutic implications of the *P. yoelii* model

Aspect	Description	References
Genetic manipulation	*P. yoelii* allows researchers to manipulate specific genes related to immune evasion or pathogenicity, facilitating the dissection of complex interactions and identifying critical virulence determinants	[63, 64]
In vivo studies	Using rodent models enables real-time observation of host-parasite interactions, allowing researchers to study immune responses and evaluate therapeutic interventions in a natural context	[65]
Comparative research	Comparative studies between *P. yoelii* and human-infecting *Plasmodium* species, such as *P. falciparum*, enable the translation of findings to human malaria research, enhancing understanding of standard and unique parasite mechanisms	[66, 67]
Targeting developmental stages	Understanding the timing of *P. yoelii* life cycle stages can inform the development of targeted interventions, potentially enhancing the effectiveness of current antimalarial therapies	[5, 68]
Optimising treatment protocols	Research into parasite chronobiology can guide the development of treatment protocols that align with the natural rhythms of both the parasite and the host, improving treatment outcomes	[69, 70]

This table highlights the advantages of *P. yoelii* in experimental research and its implications for improving antimalarial treatments

## Progress in vaccine development using *Plasmodium yoelii*

The rodent malaria parasite *P. yoelii* is particularly valuable because it mimics certain aspects of human malaria, allowing researchers to explore vaccine candidates in a controlled environment, making it a key player in understanding how the immune system responds to malaria infections [71]. One exciting area of research is the development of liver-stage vaccines [72]. When sporozoites of *P. yoelii* are introduced into laboratory mice, they can stimulate strong immune responses. Notably, according to Labaied et al. [73], *P. yoelii* parasites lacking both P52 and P36 genes exhibit normal blood-stage and mosquito-stage development but fail to progress in the liver stage. These genetically modified parasites can invade hepatocytes but cannot form the parasitophorous vacuole (PV), leading to developmental arrest in the liver and an inability to cause blood-stage infections [73]. Thus, mice immunised with these parasites were fully protected against future infections, offering a promising avenue for malaria vaccine development. This research highlights the potential of two-locus gene deletion-attenuated parasites as a foundation for creating effective *P. falciparum* live attenuated vaccines.

Similarly, studies have shown that vaccination with irradiated sporozoites can protect against later infections with live sporozoites [74–76]. According to the study conducted by Voza et al. [77], where they explored intradermal (ID) immunisation potential with attenuated *P. yoelii* sporozoites to offer comparable immunity. Mice receiving ID immunisations with additional techniques, like tape-stripping to enhance immune response, achieved protection rates of 94%, similar to IV immunisation. The results suggest that ID immunisation can be highly effective, offering a promising alternative to IV delivery. Further research is needed to identify adjuvants that reduce the number of sporozoites required for robust protective immunity, particularly for human vaccines. This finding underscores the potential of liver-stage vaccines to interrupt the malaria life cycle before the parasite reaches the bloodstream [30].

In addition to liver-stage approaches, *P. yoelii* is instrumental in exploring blood-stage vaccines [78]. Researchers have identified various antigens that trigger immune responses during this stage of infection [79–81]. For example, immunisation with the merozoite surface protein 1 (PyMSP1) has shown promise, as it led to significant reductions in parasitemia when mice were later exposed to *P. yoelii*[81]. Also, researchers purified apical merozoite antigen 1 (AMA-1) from *P. yoelii yoelii*-infected red blood cells, which induced a strong protective immune response in mice. Monoclonal antibodies specific to *P. yoelii*AMA-1 were developed, and one showed high effectiveness in combating the parasite through passive immunisation. A second protein associated with AMA-1, possibly in the rhoptry organelles, was also identified. This highlights the antigen’s potential as a vaccine candidate [80]. Moreover, *P. yoelii* helps us understand the role of CD8+T cells in malaria control. Vaccination strategies that boost the activation of these T cells have shown improved protection against *P. yoelii* infection, providing valuable insights for developing effective T-cell-based vaccines [82].

Research on *P. yoelii* has been pivotal in advancing vaccine development, unveiling the parasite’s crucial protective antigens and immune evasion strategies. Identifying potent immune response-inducing antigens, such as AMA-1, presents promising avenues for creating more effective malaria vaccines. Furthermore, studying *P. yoelii’s* immune evasion tactics provides insights into developing strategies to target critical stages of the parasite’s life cycle or enhance the host’s immunity. These findings are instrumental in formulating vaccines with enhanced protection against malaria. Table 5 provides an overview of potential vaccine candidates discovered from *P. yoelii* research.

**Table 5:** Potential vaccine candidates derived from *P. yoelii* studies

Vaccine candidate	Type of immune response	Stage of life cycle targeted	References
Circumsporozoite Protein (CSP)	Humoral immunity	Sporozoite stage	[83]
Merozoite Surface Protein 1 (MSP1)	Cellular and humoral immunity	Merozoite stage	[81]
Apical Membrane Antigen 1 (AMA1)	Cellular immunity	Merozoite stage	[80]
Blood-Stage Antigen 5 (BSA5)	Humoral immunity	Blood stage	[79]

## Genetic studies leverages advanced genetic tools to identify critical malaria-driving factors

Genetic studies of *P. yoelii* have opened exciting avenues for understanding malaria at a molecular level [84, 85]. With advancements in genetic manipulation techniques, particularly CRISPR/Cas9, researchers can delve into the intricate details of gene function, pathogenesis, and the mechanisms behind drug resistance [59, 86]. Zhang et al. [59] explored the functional roles of the *P. yoelii*ApiAP2 (PyApiAP2) gene family, which is crucial for malaria parasite development. Of 26 PyApiAP2 genes, 24 were selected for disruption, and 12 were successfully knocked out using CRISPR-Cas9. Ten genes are essential for developing male and female gametocytes, oocysts, and sporozoites. Additionally, protein expression analyses for seven PyApiAP2 gene products provided valuable insights into their functions. This systematic characterisation enhances our understanding of gene expression regulation in malaria parasites, offering potential targets for disease control and prevention strategies.

One of the standout features of *P. yoelii* is its susceptibility to genetic manipulation, which allows scientists to create targeted gene knockouts or modifications [16]. This capability enables the exploration of specific genes’ roles in the parasite’s lifecycle and how they influence the host’s immune response. For example, research by Ishizaki et al. [87] successfully characterised the pseudokinase PypPK1 in *P. yoelii*, which is highly expressed in schizonts and male gametocytes. Transgenic parasites lacking PypPK1 (ΔpPK1), generated using the CRISPR/Cas9 method, exhibited significant growth defects and reduced virulence in mice with impaired erythrocyte invasion efficiency. While gametocyte development and egress were unaffected, ΔpPK1 parasites showed significantly reduced ex-flagellation centres and oocyst formation, highlighting the crucial role of PypPK1 in both erythrocyte invasion and sexual stage development. The results demonstrated a significant reduction in invasion efficiency, shedding light on potential targets for future therapeutic interventions [87].

Moreover, manipulating genes in *P. yoelii* aids in studying drug resistance mechanisms [20]. A study by Ferrer- Rodriguez et al. [50] focused on genes linked to artemisinin resistance in the *P. yoelii* model. They investigated the mechanisms of drug resistance in *P. yoelii* by examining various parasite lines with distinct resistance profiles. The *pymdr1* gene, analogous to *pfmdr1* in *P. falciparum*, was identified and characterised, revealing a two- to three-fold increase in gene copy number in artemisinin-resistant *P. yoelii* lines compared to the parental line. By modifying these genes, the researchers could observe changes in drug susceptibility, providing insights into how resistance develops and persists, which is crucial for developing strategies to overcome drug resistance in human malaria.

The genetic studies conducted on *P. yoelii* also pave the way for investigating the pathways involved in parasite pathogenesis [35]. For instance, Witkowski et al. [47] reported that long-term in vivo selection has resulted in a murine malaria model resistant to artemisinins, revealing that resistance is linked to alterations in heme metabolism and reduced hemozoin formation due to down-expression of the Heme Detoxification Protein (HDP). Additionally, these resistant strains can detoxify free heme via a glutathione-mediated pathway, and artemisinins also inhibit hemozoin production similarly to quinolines, elucidating the mechanisms of resistance and action of these compounds. By elucidating the functions of various genes, researchers can identify potential biomarkers for disease severity or targets for vaccine development. Hence, manipulating genes involved in the parasite’s life cycle stages could inform new approaches to vaccine design, ultimately enhancing vaccine efficacy [88]. Therefore, genetic studies using *P yoelii* through techniques like CRISPR/Cas9 are invaluable for advancing our understanding of malaria. By investigating gene function and the underlying mechanisms of pathogenesis and drug resistance, researchers are better equipped to develop innovative strategies for combating this devastating disease.

## Challenges in studying *Plasmodium yoelii*

Studying *Plasmodium yoelii* as a malaria model has several challenges that limit its applicability and complicate research outcomes. One major challenge is the incomplete understanding of the parasite’s virulence mechanisms. Research shows that immune responses differ between the lethal (17XL) and non-lethal (17XNL) strains of *P. yoelii*, but the exact molecular pathways driving these differences are not fully elucidated [35]. Additionally, while *P. yoelii* is used to model drug resistance, pinpointing how resistance evolves remains difficult. For example, a two- to three-fold increase in pymdr1 gene copy number in artemisinin-resistant *P. yoelii* parallels similar findings in *P. falciparum*, but more genetic studies are required to characterise this resistance mechanism fully [89].

Host–pathogen interactions add complexity, mainly when immune responses are irregular, as seen in cases of *P. yoelii*-induced TGF-β production, which can either help clear or exacerbate infections depending on timing [90]. Furthermore, *P. yoelii* infections in mice do not always translate to human malaria models, limiting the relevance of findings to human disease [5]. Environmental factors, genetic diversity in mouse models, and co-infections also contribute to variability in research outcomes [91]. These challenges underscore the need for better models and resources to advance the understanding of malaria pathogenesis and drug resistance.

## Conclusion

*Plasmodium yoelii* has proven to be a valuable model for advancing our understanding of malaria, particularly in drug resistance, immune responses, and parasite development. However, significant challenges remain. The complexity of host-parasite interactions, variability in immune responses, and difficulties in fully translating findings to human malaria hinder the broader applicability of this model. Despite these limitations, *P. yoelii* continues to offer essential insights into malaria biology, particularly in areas where parallels can be drawn with human infections, such as gene expression regulation and the mechanisms of antimalarial drug resistance. Addressing this model’s limitations, refining experimental approaches, and integrating findings with human studies will be crucial to unlocking new therapeutic strategies and improving our overall understanding of malaria pathogenesis. Through this continued research, we can hope to make significant strides in the fight against malaria.

## Data Availability

No datasets were generated or analysed during the current study.

## References

[1] Varo R, Chaccour C, Bassat Q (2020) Update on malaria. Med Clín (English Edition) 155:395–402. 10.1016/j.medcle.2020.05.024 10.1016/j.medcli.2020.05.010 32620355

[2] Sato S (2021) Plasmodium—a brief introduction to the parasites causing human malaria and their basic biology. J Physiol Anthropol 40:1. 10.1186/s40101-020-00251-9 33413683 10.1186/s40101-020-00251-9 PMC7792015

[3] Kolawole E, Ayeni E, Abolade S, Ugwu S, Awoyinka T, Ofeh A, Okolo B (2022) Malaria endemicity in Sub-Saharan Africa: past and present issues in public health. Microbes Infectious Dis. 10.21608/mid.2022.150194.1346

[4] Oriero EC, Amenga-Etego L, Ishengoma DS, Amambua-Ngwa A (2021) *Plasmodium malariae,* current knowledge and future research opportunities on a neglected malaria parasite species. Crit Rev Microbiol 47:44–56. 10.1080/1040841X.2020.1838440 33507842 10.1080/1040841X.2020.1838440

[5] Gozalo AS, Robinson CK, Holdridge J, Franco Mahecha OL, Elkins WR (2024) Overview of *Plasmodium* spp. and animal models in malaria research. Comp Med 74:205–230. 10.30802/AALAS-CM-24-000019 38902006 10.30802/AALAS-CM-24-000019 PMC11373680

[6] Rex DAB, Patil AH, Modi PK, Kandiyil MK, Kasaragod S, Pinto SM, Tanneru N, Sijwali PS, Prasad TSK (2022) Dissecting *Plasmodium yoelii* pathobiology: proteomic approaches for decoding novel translational and post-translational modifications. ACS Omega 7:8246–8257. 10.1021/acsomega.1c03892 35309442 10.1021/acsomega.1c03892 PMC8928344

[7] Pattaradilokrat S, Wu J, Xu F, Su X (2022) The origins, isolation, and biological characterization of rodent malaria parasites. Parasitol Int 91:102636. 10.1016/j.parint.2022.102636 35926694 10.1016/j.parint.2022.102636 PMC9465976

[8] Yoeli M, Most H, Bone G (1979) *Plasmodium berghei*: cyclical transmissions by experimentally infected *Anopheles quadrimaculatus*. Science 144(1964):1580–1581. 10.1126/science.144.3626.1580 10.1126/science.144.3626.1580 14169345

[9] Knowles G, Sanderson A, Walliker D (1981) *Plasmodium yoelii*: genetic analysis of crosses between two rodent malaria subspecies. Exp Parasitol 52:243–247. 10.1016/0014-4894(81)90079-5 7274370 10.1016/0014-4894(81)90079-5

[10] Tarun AS, Peng X, Dumpit RF, Ogata Y, Silva-Rivera H, Camargo N, Daly TM, Bergman LW, Kappe SHI (2008) A combined transcriptome and proteome survey of malaria parasite liver stages. Proc Natl Acad Sci 105:305–310. 10.1073/pnas.0710780104 18172196 10.1073/pnas.0710780104 PMC2224207

[11] Schoch CL, Ciufo S, Domrachev M, Hotton CL, Kannan S, Khovanskaya R, Leipe D, Mcveigh R, O’Neill K, Robbertse B, Sharma S, Soussov V, Sullivan JP, Sun L, Turner S, Karsch-Mizrachi I, Taxonomy NCBI (2020) a comprehensive update on curation, resources and tools. Database. 10.1093/database/baaa062 10.1093/database/baaa062 PMC7408187 32761142

[12] Charoenvit Y, Mellouk S, Sedegah M, Toyoshima T, Leef MF, Delavega P, Beaudoin RL, Aikawa M, Fallarme V, Hoffman SL (1995) *Plasmodium yoelii*: 17-kDa hepatic and erythrocytic stage protein is the target of an inhibitory monoclonal antibody. Exp Parasitol 80:419–429. 10.1006/expr.1995.1054 7729477 10.1006/expr.1995.1054

[13] Li J, Zhang Y, Liu S, Hong L, Sullivan M, McCutchan TF, Carlton JM, Su X (2009) Hundreds of microsatellites for genotyping *Plasmodium yoelii* parasites. Mol Biochem Parasitol 166:153–158. 10.1016/j.molbiopara.2009.03.011 19450732 10.1016/j.molbiopara.2009.03.011 PMC2787103

[14] Deharo E, Gautret P, Ginsburg H, Chabaud AG, Landau I (1994) Synchronization of *Plasmodium yoelii* nigeriensis and *P. y. killicki* infection in the mouse by means of Percoll-glucose gradient stage fractionation: determination of the duration of the schizogonic cycle. Parasitol Res 80:159–164. 10.1007/BF00933785 8202457 10.1007/BF00933785

[15] Zhang C, Oguz C, Huse S, Xia L, Wu J, Peng Y-C, Smith M, Chen J, Long CA, Lack J, Su X (2021) Genome sequence, transcriptome, and annotation of rodent malaria parasite *Plasmodium yoelii* nigeriensis N67. BMC Genom 22:303. 10.1186/s12864-021-07555-9 10.1186/s12864-021-07555-9 PMC8072299 33902452

[16] Mota MM, Thathy V, Nussenzweig RS, Nussenzweig V (2001) Gene targeting in the rodent malaria parasite *Plasmodium yoelii*. Mol Biochem Parasitol 113:271–278. 10.1016/S0166-6851(01)00228-6 11295181 10.1016/s0166-6851(01)00228-6

[17] Khan SM, Jarra W, Preiser PR (2001) The 235 kDa rhoptry protein of *Plasmodium* (yoelii) *yoelii*: function at the junction. Mol Biochem Parasitol 117:1–10. 10.1016/S0166-6851(01)00333-4 11551627 10.1016/s0166-6851(01)00333-4

[18] Lin J, Reid AJ, Cunningham D, Böhme U, Tumwine I, Keller-Mclaughlin S, Sanders M, Berriman M, Langhorne J (2018) Genomic and transcriptomic comparisons of closely related malaria parasites differing in virulence and sequestration pattern. Wellcome Open Res 3:142. 10.12688/wellcomeopenres.14797.2 30542666 10.12688/wellcomeopenres.14797.1 PMC6259598

[19] Carlton JM, Angiuoli SV, Suh BB, Kooij TW, Pertea M, Silva JC, Ermolaeva MD, Allen JE, Selengut JD, Koo HL, Peterson JD, Pop M, Kosack DS, Shumway MF, Bidwell SL, Shallom SJ, van Aken SE, Riedmuller SB, Feldblyum TV, Cho JK, Quackenbush J, Sedegah M, Shoaibi A, Cummings LM, Florens L, Yates JR, Raine JD, Sinden RE, Harris MA, Cunningham DA, Preiser PR, Bergman LW, Vaidya AB, van Lin LH, Janse CJ, Waters AP, Smith HO, White OR, Salzberg SL, Venter JC, Fraser CM, Hoffman SL, Gardner MJ, Carucci DJ (2002) Genome sequence and comparative analysis of the model rodent malaria parasite *Plasmodium yoelii* yoelii. Nature 419:512–519. 10.1038/nature01099 12368865 10.1038/nature01099

[20] Pattaradilokrat S, Cheesman SJ, Carter R (2008) Congenicity and genetic polymorphism in cloned lines derived from a single isolate of a rodent malaria parasite. Mol Biochem Parasitol 157:244–247. 10.1016/j.molbiopara.2007.10.011 18068827 10.1016/j.molbiopara.2007.10.011

[21] Azcárate IG, Marín-García P, Kamali AN, Pérez-Benavente S, Puyet A, Diez A, Bautista JM (2014) Differential immune response associated to malaria outcome is detectable in peripheral blood following *Plasmodium yoelii* infection in mice. PLoS ONE 9:e85664. 10.1371/journal.pone.0085664 24465641 10.1371/journal.pone.0085664 PMC3900426

[22] Carter R (1978) Studies on enzyme variation in the murine malaria parasites *Plasmodium berghei*, *P. yoelii*, *P. vinckei* and *P. chabaudi* by starch gel electrophoresis. Parasitology 76:241–267. 10.1017/S0031182000048137 351525 10.1017/s0031182000048137

[23] Nair SC, Pattaradilokrat S, Zilversmit MM, Dommer J, Nagarajan V, Stephens MT, Xiao W, Tan JC, Su X (2014) Genome-wide polymorphisms and development of a microarray platform to detect genetic variations in *Plasmodium yoelii*. Mol Biochem Parasitol 194:9–15. 10.1016/j.molbiopara.2014.03.006 24685548 10.1016/j.molbiopara.2014.03.006 PMC4052438

[24] Kooij TWA, Carlton JM, Bidwell SL, Hall N, Ramesar J, Janse CJ, Waters AP (2005) A plasmodium whole-genome synteny map: indels and synteny breakpoints as foci for species-specific genes. PLoS Pathog 1:e44. 10.1371/journal.ppat.0010044 16389297 10.1371/journal.ppat.0010044 PMC1317653

[25] Williams CT, Azad AF (2010) Transcriptional analysis of the pre-erythrocytic stages of the rodent malaria parasite *Plasmodium yoelii*. PLoS One 5:e10267. 10.1371/journal.pone.0010267 20422005 10.1371/journal.pone.0010267 PMC2858153

[26] Peng Y, Qi Y, Zhang C, Yao X, Wu J, Pattaradilokrat S, Xia L, Tumas KC, He X, Ishizaki T, Qi C-F, Holder AA, Myers TG, Long CA, Kaneko O, Li J, Su X (2020) *Plasmodium yoelii* erythrocyte-binding-like protein modulates host cell membrane structure, immunity, and disease severity. MBio. 10.1128/mBio.02995-19 31911494 10.1128/mBio.02995-19 PMC6946805

[27] Otun SO, Graca R, Onisuru O, Achilonu I (2024) Evaluation of *Plasmodium berghei* models in malaria research. J Cell Signal 5:96–113

[28] Siddiqui AJ, Bhardwaj J, Goyal M, Prakash K, Adnan M, Alreshidi MM, Patel M, Soni A, Redman W (2020) Immune responses in liver and spleen against *Plasmodium yoelii* pre-erythrocytic stages in Swiss mice model. J Adv Res 24:29–41. 10.1016/j.jare.2020.02.016 32181014 10.1016/j.jare.2020.02.016 PMC7063113

[29] Mauduit M, Grüner AC, Tewari R, Depinay N, Kayibanda M, Chavatte J-M, Franetich J-F, Crisanti A, Mazier D, Snounou G, Rénia L (2009) A role for immune responses against non-CS components in the cross-species protection induced by immunization with irradiated malaria sporozoites. PLoS ONE 4:e7717. 10.1371/journal.pone.0007717 19890387 10.1371/journal.pone.0007717 PMC2766644

[30] Doolan D, Martinez-Alier N (2006) Immune response to pre-erythrocytic stages of malaria parasites. Curr Mol Med 6:169–185. 10.2174/156652406776055249 16515509 10.2174/156652406776055249

[31] Howells RE, Gilles HM, Bent NS (1985) A comparison of the pyrimethamine and cycloguanil sensitivities of the pre-erythrocytic and erythrocytic stages of drug-sensitive and -resistant strains of *Plasmodium yoelii*. Ann Trop Med Parasitol 79:247–251. 10.1080/00034983.1985.11811915 4026437 10.1080/00034983.1985.11811915

[32] Patarroyo MA, Molina-Franky J, Gómez M, Arévalo-Pinzón G, Patarroyo ME (2020) Hotspots in plasmodium and RBC receptor-ligand interactions: key pieces for inhibiting malarial parasite invasion. Int J Mol Sci 21:4729. 10.3390/ijms21134729 32630804 10.3390/ijms21134729 PMC7370042

[33] Grüber A, Gunalan K, Ramalingam JK, Manimekalai MSS, Grüber G, Preiser PR (2011) Structural characterization of the erythrocyte binding domain of the reticulocyte binding protein homologue family of *Plasmodium yoelii*. Infect Immun 79:2880–2888. 10.1128/IAI.01326-10 21482683 10.1128/IAI.01326-10 PMC3191949

[34] Lau AOT, Sacci JB, Azad AF (2001) Host responses to *Plasmodium yoelii* hepatic stages: a paradigm in host-parasite interaction. J Immunol 166:1945–1950. 10.4049/jimmunol.166.3.1945 11160243 10.4049/jimmunol.166.3.1945

[35] Xia L, Wu J, Pattaradilokrat S, Tumas K, He X, Peng Y, Huang R, Myers TG, Long CA, Wang R, Su X (2018) Detection of host pathways universally inhibited after *Plasmodium yoelii* infection for immune intervention. Sci Rep 8:15280. 10.1038/s41598-018-33599-1 30327482 10.1038/s41598-018-33599-1 PMC6191451

[36] Orengo JM, Wong KA, Ocaña-Morgner C, Rodriguez A (2008) A *Plasmodium yoelii* soluble factor inhibits the phenotypic maturation of dendritic cells. Malar J 7:254. 10.1186/1475-2875-7-254 19077314 10.1186/1475-2875-7-254 PMC2614434

[37] Sayles PC, Wassom DL (1988) Immunoregulation in murine malaria. Susceptibility of inbred mice to infection with *Plasmodium yoelii* depends on the dynamic interplay of host and parasite genes. J Immunol 141:241–248. 10.4049/jimmunol.141.1.241 3379305

[38] Zhang Y, Zhu X, Feng Y, Pang W, Qi Z, Cui L, Cao Y (2016) TLR4 and TLR9 signals stimulate protective immunity against blood-stage *Plasmodium yoelii* infection in mice. Exp Parasitol 170:73–81. 10.1016/j.exppara.2016.09.003 27646627 10.1016/j.exppara.2016.09.003

[39] Abkallo HM, Tangena J-A, Tang J, Kobayashi N, Inoue M, Zoungrana A, Colegrave N, Culleton R (2015) Within-host competition does not select for virulence in malaria parasites studies with *Plasmodium yoelii*. PLoS Pathog 11:e1004628. 10.1371/journal.ppat.1004628 25658331 10.1371/journal.ppat.1004628 PMC4450063

[40] Qi Y, Zhu F, Eastman RT, Fu Y, Zilversmit M, Pattaradilokrat S, Hong L, Liu S, McCutchan TF, Pan W, Xu W, Li J, Huang F, Su X (2015) Regulation of *Plasmodium yoelii* oocyst development by strain- and stage-specific small-subunit rRNA. MBio. 10.1128/mBio.00117-15 25759501 10.1128/mBio.00117-15 PMC4453563

[41] Omer FM, de Souza JB, Riley EM (2003) Differential induction of TGF-β regulates proinflammatory cytokine production and determines the outcome of lethal and nonlethal *Plasmodium yoelii* infections. J Immunol 171:5430–5436. 10.4049/jimmunol.171.10.5430 14607947 10.4049/jimmunol.171.10.5430

[42] Lacerda-Queiroz N, Riteau N, Eastman RT, Bock KW, Orandle MS, Moore IN, Sher A, Long CA, Jankovic D, Su X (2017) Mechanism of splenic cell death and host mortality in a *Plasmodium yoelii* malaria model. Sci Rep 7:10438. 10.1038/s41598-017-10776-2 28874800 10.1038/s41598-017-10776-2 PMC5585408

[43] Couper KN, Blount DG, Hafalla JCR, van Rooijen N, de Souza JB, Riley EM (2007) Macrophage-mediated but gamma interferon-independent innate immune responses control the primary wave of *Plasmodium yoelii* Parasitemia. Infect Immun 75:5806–5818. 10.1128/IAI.01005-07 17923512 10.1128/IAI.01005-07 PMC2168355

[44] Su X, Wu J, Xu F, Pattaradilokrat S (2022) Genetic mapping of determinants in drug resistance, virulence, disease susceptibility, and interaction of host-rodent malaria parasites. Parasitol Int 91:102637. 10.1016/j.parint.2022.102637 35926693 10.1016/j.parint.2022.102637 PMC9452477

[45] Rivera N, Ponce YM, Arán VJ, Martínez C, Malagón F (2013) Biological assay of a novel quinoxalinone with antimalarial efficacy on *Plasmodium yoelii* yoelii. Parasitol Res 112:1523–1527. 10.1007/s00436-013-3298-2 23338979 10.1007/s00436-013-3298-2

[46] Srivastava K, Agarwal P, Soni A, Puri SK (2017) Correlation between in vitro and in vivo antimalarial activity of compounds using CQ-sensitive and CQ-resistant strains of *Plasmodium falciparum* and CQ-resistant strain of P. yoelii. Parasitol Res 116:1849–1854. 10.1007/s00436-017-5455-5 28502016 10.1007/s00436-017-5455-5

[47] Witkowski B, Lelièvre J, Nicolau-Travers M-L, Iriart X, Njomnang Soh P, Bousejra-ElGarah F, Meunier B, Berry A, Benoit-Vical F (2012) Evidence for the contribution of the hemozoin synthesis pathway of the Murine *Plasmodium yoelii* to the resistance to Artemisinin-related drugs. PLoS One 7:e32620. 10.1371/journal.pone.0032620 22403683 10.1371/journal.pone.0032620 PMC3293827

[48] Zhang Q, Ao Z, Hu N, Hu X, Liao F, Han D (2021) Artemisinin–Ginkgo biloba extract combination therapy for *Plasmodium yoelii*. Parasitol Int 80:102226. 10.1016/j.parint.2020.102226 33137498 10.1016/j.parint.2020.102226

[49] Na-Bangchang K, Karbwang J (2009) Current status of malaria chemotherapy and the role of pharmacology in antimalarial drug research and development. Fundam Clin Pharmacol 23:387–409. 10.1111/j.1472-8206.2009.00709.x 19709319 10.1111/j.1472-8206.2009.00709.x

[50] Ferrer-Rodríguez I, Pérez-Rosado J, Gervais GW, Peters W, Robinson BL, Serrano AE (2004) *Plasmodium yoelii*: identification and partial characterization of an *MDR1* gene in an artemisinin-resistant line. J Parasitol 90:152–160. 10.1645/GE-3225 15040683 10.1645/GE-3225

[51] Dutta G, Bajpai R, Vishwakarma R (1989) Antimalarial efficacy of arteether against multiple drug resistant strain of *Plasmodium yoelii* nigeriensis. Pharmacol Res 21:415–419. 10.1016/1043-6618(89)90159-X 2771860 10.1016/1043-6618(89)90159-x

[52] Ch’ng J-H, Lee Y-Q, Gun SY, Chia W-N, Chang Z-W, Wong L-K, Batty KT, Russell B, Nosten F, Renia L, Tan KS-W (2014) Validation of a chloroquine-induced cell death mechanism for clinical use against malaria. Cell Death Dis 5:e1305–e1305. 10.1038/cddis.2014.265 24967967 10.1038/cddis.2014.265 PMC4611737

[53] Muregi FW, Kano S, Kino H, Ishih A (2009) *Plasmodium berghei*: efficacy of 5-fluoroorotate in combination with commonly used antimalarial drugs in a mouse model. Exp Parasitol 121:376–380. 10.1016/j.exppara.2009.01.009 19271282 10.1016/j.exppara.2009.01.009

[54] Baina MT, Djontu JC, Mbama Ntabi JD, Mfoutou Mapanguy CC, Lissom A, Vouvoungui CJ, Boumpoutou RK, Mouanga AM, Nguimbi E, Ntoumi F (2024) Polymorphisms in the Pfcrt, Pfmdr1, and Pfk13 genes of *Plasmodium falciparum* isolates from southern Brazzaville, Republic of Congo. Sci Rep 14:27988. 10.1038/s41598-024-78670-2 39543235 10.1038/s41598-024-78670-2 PMC11564878

[55] Tuedom AGB, Sarah-Matio EM, Moukoko CEE, Feufack-Donfack BL, Maffo CN, Bayibeki AN, Awono-Ambene HP, Ayong L, Berry A, Abate L, Morlais I, Nsango SE (2021) Antimalarial drug resistance in the Central and Adamawa regions of Cameroon: prevalence of mutations in *P. falciparum* crt, Pfmdr1, Pfdhfr and Pfdhps genes. PLoS One 16:e0256343. 10.1371/journal.pone.0256343 34411157 10.1371/journal.pone.0256343 PMC8376100

[56] Berzosa P, Esteban-Cantos A, García L, González V, Navarro M, Fernández T, Romay-Barja M, Herrador Z, Rubio JM, Ncogo P, Santana-Morales M, Valladares B, Riloha M, Benito A (2017) Profile of molecular mutations in pfdhfr, pfdhps, pfmdr1, and pfcrt genes of *Plasmodium falciparum* related to resistance to different anti-malarial drugs in the Bata District (Equatorial Guinea). Malar J 16:28. 10.1186/s12936-016-1672-0 28086777 10.1186/s12936-016-1672-0 PMC5237300

[57] Sermwittayawong N, Nishibuchi M, Sawangjaroen N, Vuddhakul V (2015) Characterization of malaria infection at two border areas of Thailand adjoining with Myanmar and Malaysia. Southeast Asian J Trop Med Public Health 46:551–557 26867373

[58] Ling IT, Kaneko O, Narum DL, Tsuboi T, Howell S, Taylor HM, Scott-Finnigan TJ, Torii M, Holder AA (2003) Characterisation of the rhoph2 gene of *Plasmodium falciparum* and *Plasmodium yoelii*. Mol Biochem Parasitol 127:47–57. 10.1016/S0166-6851(02)00302-X 12615335 10.1016/s0166-6851(02)00302-x

[59] Zhang C, Li Z, Cui H, Jiang Y, Yang Z, Wang X, Gao H, Liu C, Zhang S, Su X, Yuan J (2017) Systematic CRISPR-Cas9-mediated modifications of *Plasmodium yoelii* ApiAP2 genes reveal functional insights into parasite development. MBio. 10.1128/mBio.01986-17 29233900 10.1128/mBio.01986-17 PMC5727417

[60] Claser C, Chang ZW, Russell B, Rénia L (2017) Adaptive immunity is essential in preventing recrudescence of *Plasmodium yoelii* malaria parasites after artesunate treatment. Cell Microbiol 19:e12763. 10.1111/cmi.12763 10.1111/cmi.12763 28664674

[61] Rasmussen C, Alonso P, Ringwald P (2022) Current and emerging strategies to combat antimalarial resistance. Expert Rev Anti Infect Ther 20:353–372. 10.1080/14787210.2021.1962291 34348573 10.1080/14787210.2021.1962291

[62] Parra M, Yang J, Weitner M, Akkoyunlu M (2021) Neonatal mice resist *Plasmodium yoelii* infection until exposed to para-aminobenzoic acid containing diet after weaning. Sci Rep 11:90. 10.1038/s41598-020-79703-2 33420157 10.1038/s41598-020-79703-2 PMC7794322

[63] Janse CJ, Franke-Fayard B, Mair GR, Ramesar J, Thiel C, Engelmann S, Matuschewski K, van Gemert GJ, Sauerwein RW, Waters AP (2006) High efficiency transfection of *Plasmodium berghei* facilitates novel selection procedures. Mol Biochem Parasitol 145:60–70. 10.1016/j.molbiopara.2005.09.007 16242190 10.1016/j.molbiopara.2005.09.007

[64] Nair SC, Xu R, Pattaradilokrat S, Wu J, Qi Y, Zilversmit M, Ganesan S, Nagarajan V, Eastman RT, Orandle MS, Tan JC, Myers TG, Liu S, Long CA, Li J, Su X (2017) A *Plasmodium yoelii* HECT-like E3 ubiquitin ligase regulates parasite growth and virulence. Nat Commun 8:223. 10.1038/s41467-017-00267-3 28790316 10.1038/s41467-017-00267-3 PMC5548792

[65] Khan AA, Taylor MC, Fortes Francisco A, Jayawardhana S, Atherton RL, Olmo F, Lewis MD, Kelly JM (2024) Animal models for exploring Chagas disease pathogenesis and supporting drug discovery. Clin Microbiol Rev. 10.1128/cmr.00155-23 39545730 10.1128/cmr.00155-23 PMC11629624

[66] Joste V, Guillochon E, Clain J, Coppée R, Houzé S (2022) Development and optimization of a selective whole-genome amplification to study *Plasmodium ovale* Spp. Microbiol Spectr. 10.1128/spectrum.00726-22 36098524 10.1128/spectrum.00726-22 PMC9602584

[67] Quansah E, Chen Y, Yang S, Wang J, Sun D, Zhao Y, Chen M, Yu L, Zhang C (2023) CRISPR-Cas13 in malaria parasite: diagnosis and prospective gene function identification. Front Microbiol. 10.3389/fmicb.2023.1076947 36760507 10.3389/fmicb.2023.1076947 PMC9905151

[68] Vijayan K, Wei L, Glennon EKK, Mattocks C, Bourgeois N, Staker B, Kaushansky A (2021) Host-targeted interventions as an exciting opportunity to combat malaria. Chem Rev 121:10452–10468. 10.1021/acs.chemrev.1c00062 34197083 10.1021/acs.chemrev.1c00062 PMC12677007

[69] Prior KF, Middleton B, Owolabi ATY, Westwood ML, Holland J, O’Donnell AJ, Blackman MJ, Skene DJ, Reece SE (2021) Synchrony between daily rhythms of malaria parasites and hosts is driven by an essential amino acid. Wellcome Open Res 6:186. 10.12688/wellcomeopenres.16894.2 34805551 10.12688/wellcomeopenres.16894.1 PMC8577053

[70] O’Donnell AJ, Reece SE (2021) Ecology of asynchronous asexual replication: the intraerythrocytic development cycle of *Plasmodium berghei* is resistant to host rhythms. Malar J 20:105. 10.1186/s12936-021-03643-z 33608011 10.1186/s12936-021-03643-z PMC7893937

[71] Zheng L, Pang W, Qi Z, Luo E, Cui L, Cao Y (2016) Effects of transmission-blocking vaccines simultaneously targeting pre- and post-fertilization antigens in the rodent malaria parasite *Plasmodium yoelii*. Parasit Vectors 9:433. 10.1186/s13071-016-1711-2 27502144 10.1186/s13071-016-1711-2 PMC4977633

[72] Alaro JR, Lynch MM, Burns JM (2010) Protective immune responses elicited by immunization with a chimeric blood-stage malaria vaccine persist but are not boosted by *Plasmodium yoelii* challenge infection. Vaccine 28:6876–6884. 10.1016/j.vaccine.2010.08.018 20709001 10.1016/j.vaccine.2010.08.018 PMC2948860

[73] Labaied M, Harupa A, Dumpit RF, Coppens I, Mikolajczak SA, Kappe SHI (2007) *Plasmodium yoelii* sporozoites with simultaneous deletion of P52 and P36 are completely attenuated and confer sterile immunity against infection. Infect Immun 75:3758–3768. 10.1128/IAI.00225-07 17517871 10.1128/IAI.00225-07 PMC1951999

[74] Good M (2005) Vaccine-induced immunity to malaria parasites and the need for novel strategies. Trends Parasitol 21:29–34. 10.1016/j.pt.2004.10.006 15639738 10.1016/j.pt.2004.10.006

[75] Leitner WW, Bergmann-Leitner ES, Angov E (2010) Comparison of *Plasmodium berghei* challenge models for the evaluation of pre-erythrocytic malaria vaccines and their effect on perceived vaccine efficacy. Malar J 9:145. 10.1186/1475-2875-9-145 20507620 10.1186/1475-2875-9-145 PMC2904356

[76] Rajneesh R, Tiwari VK, Singh A, Kumar RP, Gupta AK, Singh V, Gautam R (2023) Kumar, advancements and challenges in developing malaria vaccines: targeting multiple stages of the parasite life cycle. ACS Infect Dis 9:1795–1814. 10.1021/acsinfecdis.3c00332 37708228 10.1021/acsinfecdis.3c00332

[77] Voza T, Kebaier C, Vanderberg JP (2010) Intradermal immunization of mice with radiation-attenuated sporozoites of *Plasmodium yoelii* induces effective protective immunity. Malar J 9:362. 10.1186/1475-2875-9-362 21159170 10.1186/1475-2875-9-362 PMC3014973

[78] Huang X, Liew K, Natalang O, Siau A, Zhang N, Preiser PR (2013) The role of serine-type serine repeat antigen in *Plasmodium yoelii* blood stage development. PLoS ONE 8:e60723. 10.1371/journal.pone.0060723 23634205 10.1371/journal.pone.0060723 PMC3636278

[79] Burns JM, Dunn PD, Russo DM (1997) Protective immunity against *Plasmodium yoelii* malaria induced by immunization with particulate blood-stage antigens. Infect Immun 65:3138–3145. 10.1128/iai.65.8.3138-3145.1997 9234766 10.1128/iai.65.8.3138-3145.1997 PMC175443

[80] Narum DL, Ogun SA, Thomas AW, Holder AA (2000) Immunization with parasite-derived apical membrane antigen 1 or passive immunization with a specific monoclonal antibody protects BALB/c mice against lethal *Plasmodium yoelii yoelii* YM blood-stage infection. Infect Immun 68:2899–2906. 10.1128/IAI.68.5.2899-2906.2000 10768987 10.1128/iai.68.5.2899-2906.2000 PMC97502

[81] Ahlborg N, Ling IT, Howard W, Holder AA, Riley EM (2002) Protective Immune responses to the 42-kilodalton (kDa) region of *Plasmodium yoelii* merozoite surface protein 1 are induced by the C-terminal 19-kDa region but not by the adjacent 33-kDa region. Infect Immun 70:820–825. 10.1128/IAI.70.2.820-825.2002 11796616 10.1128/IAI.70.2.820-825.2002 PMC127676

[82] Chandele A, Mukerjee P, Das G, Ahmed R, Chauhan VS (2011) Phenotypic and functional profiling of malaria-induced CD8 and CD4 T cells during blood-stage infection with *Plasmodium yoelii*. Immunology 132:273–286. 10.1111/j.1365-2567.2010.03363.x 21039472 10.1111/j.1365-2567.2010.03363.x PMC3050450

[83] Zhang M, Kaneko I, Tsao T, Mitchell R, Nardin EH, Iwanaga S, Yuda M, Tsuji M (2016) A highly infectious *Plasmodium yoelii* parasite, bearing *Plasmodium falciparum* circumsporozoite protein. Malar J 15:201. 10.1186/s12936-016-1248-z 27068454 10.1186/s12936-016-1248-z PMC4828769

[84] Vaughan A, Chiu S-Y, Ramasamy G, Li L, Gardner MJ, Tarun AS, Kappe SHI, Peng X (2008) Assessment and improvement of the *Plasmodium yoelii yoelii* genome annotation through comparative analysis. Bioinformatics 24:i383–i389. 10.1093/bioinformatics/btn140 18586738 10.1093/bioinformatics/btn140 PMC2718618

[85] Li J, Pattaradilokrat S, Zhu F, Jiang H, Liu S, Hong L, Fu Y, Koo L, Xu W, Pan W, Carlton JM, Kaneko O, Carter R, Wootton JC, Su X (2011) Linkage maps from multiple genetic crosses and loci linked to growth-related virulent phenotype in *Plasmodium yoelii*. Proceed Natl Acad Sci. 10.1073/pnas.1102261108 10.1073/pnas.1102261108 PMC3150948 21690382

[86] Zhang C, Gao H, Yang Z, Jiang Y, Li Z, Wang X, Xiao B, Su X, Cui H, Yuan J (2017) CRISPR/Cas9 mediated sequential editing of genes critical for ookinete motility in *Plasmodium yoelii*. Mol Biochem Parasitol 212:1–8. 10.1016/j.molbiopara.2016.12.010 28034675 10.1016/j.molbiopara.2016.12.010 PMC5580835

[87] Ishizaki T, Chaiyawong N, Hakimi H, Asada M, Tachibana M, Ishino T, Yahata K, Kaneko O (2020) A novel *Plasmodium yoelii* pseudokinase, PypPK1, is involved in erythrocyte invasion and exflagellation center formation. Parasitol Int 76:102056. 10.1016/j.parint.2020.102056 31953169 10.1016/j.parint.2020.102056

[88] Kreutzfeld O, Müller K, Matuschewski K (2017) Engineering of genetically arrested parasites (GAPs) for a precision malaria vaccine. Front Cell Infect Microbiol. 10.3389/fcimb.2017.00198 28620583 10.3389/fcimb.2017.00198 PMC5450620

[89] Witkowski B, Berry A, Benoit-Vical F (2009) Resistance to antimalarial compounds: methods and applications. Drug Resist Updates 12:42–50. 10.1016/j.drup.2009.01.001 10.1016/j.drup.2009.01.001 19285915

[90] Burgess V, Maya JD (2023) Statin and aspirin use in parasitic infections as a potential therapeutic strategy: a narrative review. Rev Argent Microbiol 55:278–288. 10.1016/j.ram.2023.01.006 37019801 10.1016/j.ram.2023.01.006

[91] Knowles SCL (2011) The effect of helminth co-infection on malaria in mice: a meta-analysis. Int J Parasitol 41:1041–1051. 10.1016/j.ijpara.2011.05.009 21777589 10.1016/j.ijpara.2011.05.009

